# Overexpression of GM3 and Ganglioside Pattern Remodeling in Lung Adenocarcinoma Brain Metastases Identified by Ion Mobility Mass Spectrometry

**DOI:** 10.3390/ijms262412029

**Published:** 2025-12-14

**Authors:** Mirela Sarbu, Raluca Ica, Željka Vukelić, David E. Clemmer, Alina D. Zamfir

**Affiliations:** 1 Department of Condensed Matter, National Institute for Research and Development in Electrochemistry and Condensed Matter, 300224 Timisoara, Romania; mirela.sarbu86@yahoo.co.uk (M.S.); raluca.ica@gmail.com (R.I.); 2 Department of Technical and Natural Sciences, “Aurel Vlaicu” University of Arad, 310130 Arad, Romania; 3 Department of Chemistry and Biochemistry, Faculty of Medicine, University of Zagreb, 10000 Zagreb, Croatia; zeljka.vukelic@mef.hr; 4 Department of Chemistry, The College of Arts & Science, Indiana University, Bloomington, IN 47405-7102, USA; clemmer@indiana.edu

**Keywords:** lung adenocarcinoma, brain metastases, ion mobility spectrometry mass spectrometry, ganglioside biomarker, ceramide metabolism

## Abstract

Lung adenocarcinoma (LUAD), the most prevalent subtype of non-small cell lung carcinoma (NSCLC), commonly metastasizes to the brain, particularly in advanced stages. Since brain metastases (BMs) are a leading cause of morbidity and mortality in LUAD patients, their early detection is critical, necessitating the identification of reliable biomarkers. Gangliosides (GGs), a class of bioactive glycosphingolipids involved in cell signaling, adhesion, and immune regulation, have emerged as promising candidates for diagnostic and therapeutic targeting in LUAD-associated brain metastases (BMLA). In this context, ion mobility spectrometry mass spectrometry (IMS-MS) was employed here to analyze GG alterations in BMLA tissues compared to healthy cerebellar control. The results revealed marked differences, including a reduction in the total number of species, altered sialylation profiles, and variations in fatty acid chain length and sphingoid base hydroxylation. GM3, a monosialodihexosylganglioside, was significantly overexpressed in BMLA, supporting its role in tumor progression via immune evasion and oncogenic signaling. Elevated levels of the brain-specific GT1 ganglioside further point to its possible role as a metastasis-associated biomarker, while the presence of asialogangliosides, absent in normal brain, suggests adaptation to the brain microenvironment. Structural modifications such as *O*-acetylation, fucosylation, and CH_3_COO^−^ were more frequent in BMLA, being associated with aggressive tumor phenotypes. Ceramide profiles revealed increased levels of proliferative C16- and C24-ceramides and decreased pro-apoptotic C18-ceramide. Additionally, GM3(d18:1/22:0) and GD3(d18:1/16:0), identified as potential BMLA biomarkers, were structurally characterized using (−) nanoelectrospray ionization (nanoESI) IMS collision-induced dissociation tandem MS (CID MS/MS). Collectively, these findings highlight the clinical potential of GGs for early diagnosis and targeted therapy in BMLA.

## 1. Introduction

Lung cancer remains the leading cause of cancer-related mortality worldwide [1] and is among the most frequent primary sources of brain metastases (BMs) [1,2]. It encompasses a heterogeneous group of malignancies, primarily classified into small cell lung carcinoma (SCLC) and non-small cell lung carcinoma (NSCLC), with the latter accounting for approximately 70–75% of cases [3,4]. Most patients are diagnosed at advanced or metastatic stages, contributing to the persistently poor prognosis [5].

Lung adenocarcinoma (LUAD) is the most prevalent histological subtype of NSCLC, comprising nearly 50% of cases. It typically originates in the distal airways, with tumorigenesis often beginning in the epithelial lining of the mucosal glands [6,7]. One of the most formidable challenges in the management of LUAD is its high propensity to metastasize, particularly to the brain. BMs occur in a significant proportion of patients, often within the first two years following diagnosis, and are associated with increased morbidity, reduced quality of life, and high mortality rates [4,8]. The metastatic cascade involves hematogenous dissemination, traversal of the blood–brain barrier (BBB), and colonization of the brain parenchyma, mediated by complex interactions between tumor cells and the neural microenvironment and influenced by tumor-intrinsic genetic alterations [9,10]. For example, *EGFR* mutations are associated with increased BM risk, particularly in non-smokers [11,12], whereas *KRAS* mutations are more common in male patients with BMs [12]. While tobacco use remains the dominant risk factor, 15–20% of LUAD cases occur in non-smokers, implicating genetic and environmental contributions.

Despite progress in therapeutic strategies, LUAD remains a major public health burden, with persistently low survival rates driven by delayed diagnosis, high metastatic potential, molecular heterogeneity, and treatment resistance [13]. The pronounced intra- and inter-tumoral heterogeneity underscores the urgent need for reliable biomarkers to support early detection, guide therapy selection, and monitor disease progression [14,15]. Brain metastases further complicate management, as the restricted permeability of the BBB and adaptive resistance mechanisms limit therapeutic efficacy. Optimal care for LUAD patients with BMs requires a multimodal approach—combining reliable biomarker identification [14,15] with targeted therapies, radiotherapy, and supportive care.

Early detection of BMs is particularly critical, yet remains challenging: while conventional imaging techniques, such as magnetic resonance imaging (MRI) [16,17,18,19] and computed tomography (CT) [16,17,20], are essential for identifying metastatic lesions, many occult micro-metastases remain evasive toearly identification. As a result, BMs are often diagnosed only after clinical symptoms emerge or imaging abnormalities become evident, leading to significant diagnostic delays [20].

To overcome these limitations, the integration of molecular biomarkers [21,22], such as gangliosides (GGs) [23], into clinical workflows may enhance diagnostic precision. GGs, a subclass of glycosphingolipids enriched in the outer leaflet of the plasma membrane, are emerging as promising targets due to their roles in cell adhesion, signaling, and immune modulation [23,24,25]. Structurally composed of ceramide linked to sialylated oligosaccharides, GGs exhibit altered expression in cancers, especially of GM3 and GD3, influencing metastatic behavior and therapeutic resistance. Notably, BMs often retain the molecular features of the primary tumor, including GG composition.

Targeting lipid-related metabolic pathways has therefore emerged as a promising strategy for both biomarker discovery and therapeutic intervention in LUAD. Tumor-associated GGs, such as GD2 and GM2, are under investigation for their dual role as therapeutic targets and diagnostic markers. Monoclonal antibodies directed against these GGs [26,27] are under evaluation for their potential to selectively eliminate LUAD cells and serve as imaging agents for early detection of metastasis. Furthermore, modulation of sphingolipid signaling—particularly through ceramide and sphingosine-1-phosphate pathways—offers a means of regulating apoptosis, proliferation, and chemoresistance [28]. Other strategies involve disrupting lipid biosynthesis and energy metabolism, notably by inhibiting fatty acid synthase [29,30,31] or carnitine palmitoyltransferase 1 [32,33], both frequently upregulated in LUAD to support aggressive tumor growth.

These advances align with the broader precision medicine paradigm, which has already transformed LUAD management through therapies targeting specific oncogenic drivers. Molecularly targeted agents, such as tyrosine kinase inhibitors (TKIs) for patients harboring *EGFR*, *ALK*, *ROS1*, or *MET* alterations [34,35], and immune checkpoint inhibitors (ICIs) targeting PD-1, PD-L1 and CTLA-4 [36,37,38] have transformed outcomes in genetically defined NSCLC populations. Immunotherapy has emerged as a first-line treatment for metastatic NSCLC [36,38], achieving durable responses in select patients, particularly those with high PD-L1 expression [39,40]. However, resistance remains a major challenge, with approximately 80% of patients exhibiting primary resistance [41] and acquired resistance frequently developing within months [41,42,43].

Complementing immunotherapy, targeted therapy focused on inhibiting oncogenic signaling cascades hasdemonstrated substantial efficacy in halting tumor progression and metastasis, while reducing toxicity compared to traditional treatments [44,45,46,47,48]. TKIs directed against *EGFR*, *ALK*, *VEGF*, and *KRAS* [34,35,49,50,51] have shown significant clinical benefits in molecularly stratified NSCLC subtypes [52], yet therapeutic resistance and tumor recurrence continue to impede long-term survival. Nanotechnology-based systems, including nanobubbles, enhance therapeutic precision and reduce toxicity, though evolving tumor resistance continues to limit long-term survival [53,54,55,56]. These limitations collectively highlight the critical need for novel biomarkers and targeted therapeutic strategies [52].

Beyond protein-based targets, GGs have gained prominence due to their roles in tumor immune evasion, adhesion, migration, and apoptosis resistance, particularly in brain metastases of lung adenocarcinoma (BMLA) [57,58,59]. In this context, our study employs advanced mass spectrometry (MS) platforms to elucidate ganglioside profiles in metastatic LUAD. High-resolution MS (HRMS), used independently or in combination with ion mobility spectrometry (IMS), has enabled precise structural characterization of GGs [60,61,62,63,64,65]. Chip-based nanoelectrospray ionization (nanoESI) MS and IMS-MS further facilitate comprehensive profiling of GG variants in tumor tissues [66,67,68,69], including glioblastomas [70,71] and astrocytomas [72]. IMS-MS discriminates GGs based on charge state, carbohydrate chain length, degree of sialylation, and ceramide composition [72,73], enabling the identification of molecular signatures associated with malignancy [70,74]. These analytical capabilities lay the groundwork for non-invasive diagnostics and precision therapeutics. Incorporating GG biomarker profiling into clinical practice may significantly improve early detection of BMLA [23], guide therapeutic monitoring, and advance personalized oncology.

Accordingly, the present study aims to identify GG biomarkers for the early diagnosis and monitoring of patients with BMLA using IMS-MS and advance personalized oncology. These findings underscore the diagnostic and therapeutic potential of GG profiling in metastatic LUAD and support the integration of IMS-MS into personalized oncology strategies.

## 2. Results

### 2.1. IMS-MS Screening of Gangliosides in Brain Metastases from Lung Adenocarcinoma

**Figure 1:**
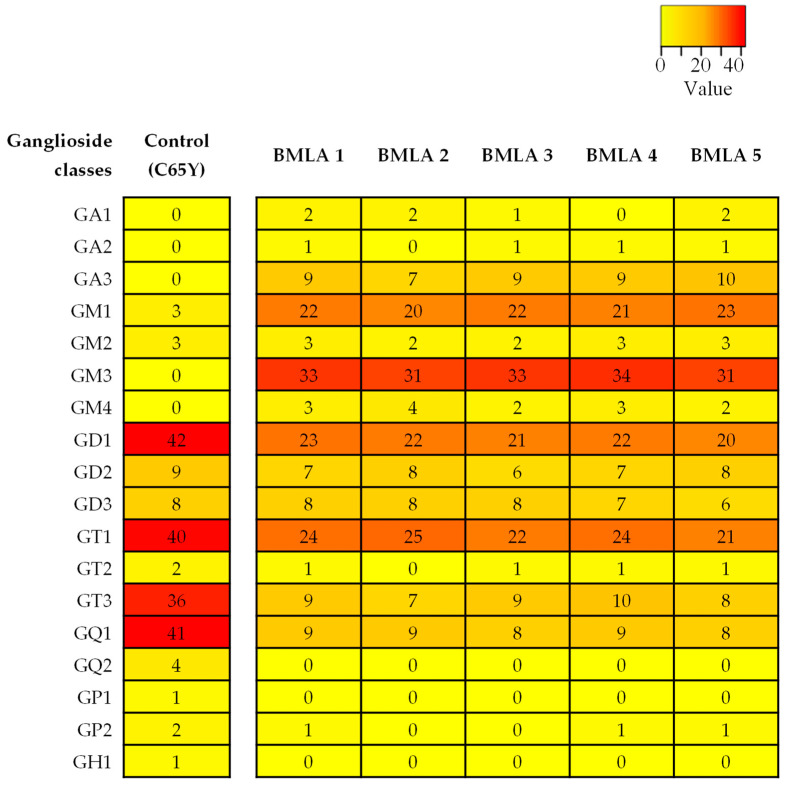
Heatmap of ganglioside expression profiles in BMLA. The heatmap illustrates the number of ganglioside (GG) species identified in five BMLA tissue samples and in the C65Y control. Each row corresponds to a GG class, and each column to a tissue sample. Numerical values inside the heatmap represent the exact count of detected GG species for the corresponding class and sample. No normalization was applied. The color gradient bar indicates the relative number of GG species identified across the dataset. Red represents classes with a higher number of detected species, while yellow represents classes with fewer detected species. Lower color intensity corresponds to lower number of identified species.

**Figure 2:**
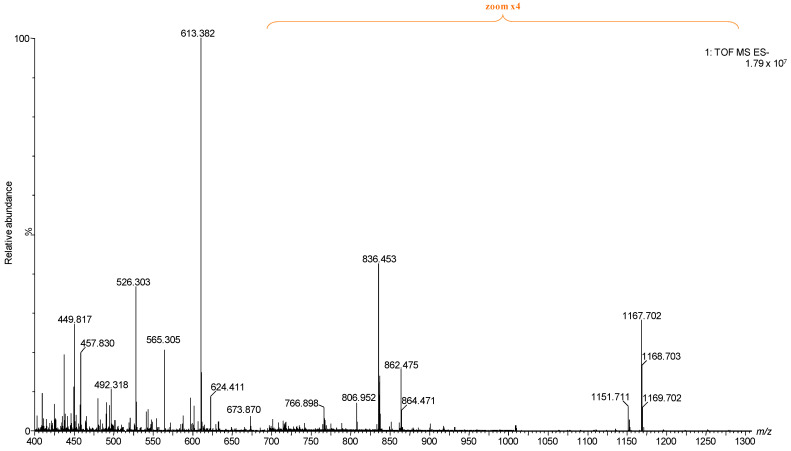
Full (−) nanoESI IMS-MS of the representative BMLA1 presented in the manuscript.

The comparative evaluation presented in Figure 1, which includes data from all five BMLA cases alongside control data from a healthy cerebellum of a 65-year-old (C65Y) male patient [73], revealed that the five BMLA samples exhibited no significant inter-sample variability in GG composition, structural features, or ion mobility profiles, while the control was included for reference. The overall GG expression patterns, including key alterations in glycan complexity, sialylation, and ceramide composition, were consistent across all BMLA samples. Because of this strong molecular similarity, and to streamline data presentation without redundancy, we elected to report detailed IMS-MS results in the manuscript from a single representative sample. This approach ensures clarity while accurately reflecting the broader molecular trends observed in the full sample set. The full scan mass spectrum of the representative BMLA sample is shown in Figure 2, while full scan spectra for the remaining four BMLA samples are provided in Supplementary Figures S1–S4.

Consistent with previous GG studies, IMS enabled the deconvolution of complex spectra by separating GG classes into distinct mobility families based on charge state, glycan chain length, Neu5Ac content, and ceramide composition. By integrating data over defined drift-time regions specific to each class, the method enabled detection and identification of a greater number of species, including those with low abundance.

**Figure 3:**
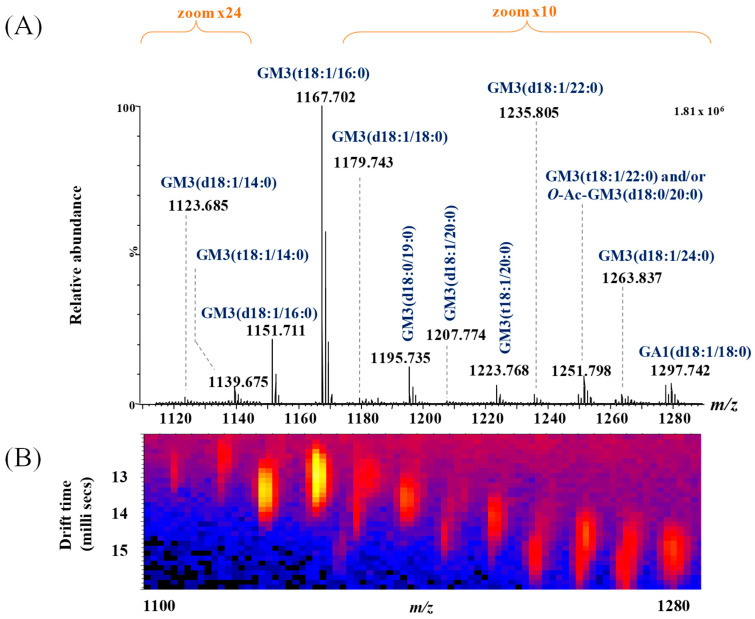
(**A**) Extracted (−) nanoESI-IMS MS corresponding to singly charged GA1 and GM3 ganglioside species, obtained from the total BMLA driftscope display (drift time versus *m*/*z*), illustrating the distribution of electrosprayed GG ions; (**B**) driftscope plot (*m*/*z* versus drift time) showing GA1 and GM3 species detected in BMLA.

**Figure 4:**
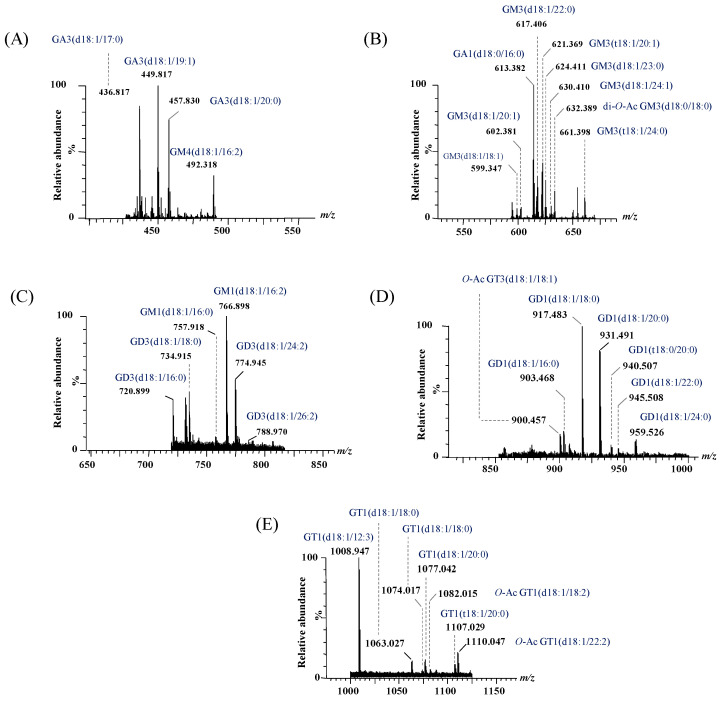
Extracted (−) nanoESI IMS mass spectra of doubly charged GG species from the total BMLA driftscope display: (**A**) GA3 and GM4, (**B**) GA1 and GM3, (**C**) GM1 and GD3, (**D**) GD1 and GT3, and (**E**) GT1.

**Figure 5:**
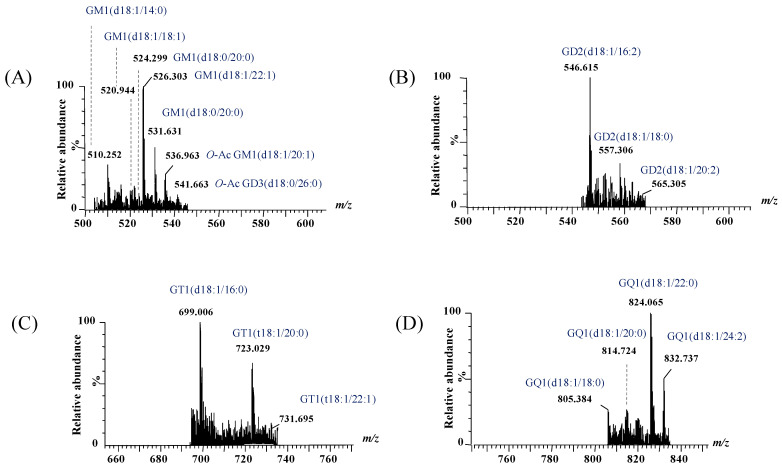
Extracted (−) nanoESI IMS mass spectra of doubly charged GG species from the total BMLA driftscope display: (**A**) GM1 and GD3, (**B**) GD2, (**C**) GT1, and (**D**) GQ1.

Representative (−) nanoESI IMS-MS spectra and driftscope displays (mass to charge ratio, *m*/*z*, versus drift time) are shown in Figure 3A,B for singly charged asialo- and monosialylated GGs, and in Figure 4 and Figure 5 for doubly and triply charged species. To facilitate structural interpretation, Table 1 summarizes the detected and identified species per ganglioside class. Full dataset including *m*/*z* values and mass accuracy (within an average mass accuracy cutoff of 6.5 ppm) is provided in Supplementary Table S1. As shown in Table S1, out of the identified ganglioside species, 165 compounds/ions (91%) exhibited a mass error within 0–10 ppm, indicating high mass accuracy and strong confidence in their identification. Only 16 ions (9%) showed a mass error above 10 ppm, which remains within the acceptable tolerance for high-resolution mass spectrometry of complex lipids, particularly in biological matrices. The species with ppm values above 10 ppm correspond to ions detected at lower *m*/*z*, where identical absolute mass deviations produce proportionally higher ppm values, a well-recognized effect in HRMS. A comprehensive analysis of Figure 3, Figure 4 and Figure 5, Table S1 and Table 1, dominated by doubly- and triply charged GGs, reveals a complex molecular pattern and the presence of biologically relevant modifications. 168 ions corresponding to 155 distinct GG species were identified, underscoring the high structural complexity of the BMLA glycosphingolipidome.

**Figure 6:**
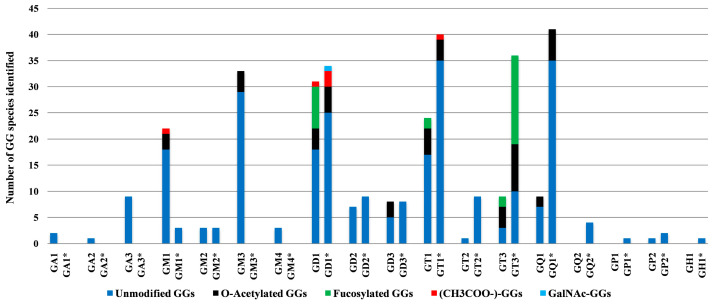
Number of GG species identified by IMS-MS in BMLA tissue and C65Y (indicated by asterisk) [73], plotted according to glycan core composition and sialylation degree.

To gain deeper insights into the alterations in GG expression associated with BMLA, Figure 6 presents the number of GG species identified in the representative BMLA case discussed in this study, alongside the C65Y control [73], using IMS-MS with a focus on glycan composition and sialylation patterns. The observed reductions in both sialylation levels and glycan chain complexity in BMLA likely result from a combination of an accelerated turnover rate of GGs and a diminished biosynthetic activity, potentially due to altered expression of specific glycosyltransferases and sialyltransferases.

Notably, while polysialylated, long-chain GGs are dominant in normal cerebellar tissue [73], BMLA tissue exhibited marked reductions in both total GG species and glycan complexity, including lower levels of Neu5Ac and shorter glycan chains. Approximately 64% of detected glycoforms were mono- and disialylated species, namely GM1, GM2, GM3, GM4, GD1, GD2, and GD3, with GM3 being the most abundant, followed by GM1 and GD1 variants with diverse ceramide backbones. In contrast, GD1, GT1, and GQ1 predominate in healthy brain tissue [73].

The IMS-MS data presented in Table 1 also emphasize the significant abundance and potential biomarker role of GT1 glycoforms, which rank second in abundance in BMLA, following GM3. A total of 17 such species bearing ceramides of varying compositions were detected and identified in BMLA through IMS-MS. Additionally, an interesting finding from IMS-MS and subsequent data interpretation was also the identification of 12 asialo GalNAcGalGlc-Cer species, i.e., two from GA1, one from GA2, and nine from GA3, highlighting a possibly unique GG signature for BMLA. Table 1 further indicates widespread peripheral modifications across several GG classes. *O*-acetylation of Neu5Ac was identified in 25 species, while fucosylation of sugar units was observed in four GT species, and additional non-carbohydrate attachments in GD1 and GM1. Highly expressed GG classes, including GM1, GM3, GD1, GD3, GT1, GT3, and GQ1, frequently carried *O*-acetyl groups, whereas GT1 and GT3 exhibited fucosylation.

Alterations in ceramide composition and sialylation patterns were consistent with known tumor-related changes in human carcinomas [75,76,77]. For example, while 22% of GGs identified in C65Y samples were associated with odd-chained fatty acids (OCFAs; C13–C31, with C17, C19, and C21 being predominant), only 5% of GGs in BMLA samples contained C17 or C19 fatty acids. This supports prior findings [23] that reduced OCFA presence is a distinguishing feature of BMLA GGs. In prior studies, C17 to C23 fatty acids were specifically associated with BMLA.

In addition to the differences in the number of species with OCFA, there was also a notable distinction in terms of glycan content. Species with low sialylation and/or shorter carbohydrate chains, such as GA1, GA2, GM3, GD1 and GD2, were found in both this and earlier [23] BMLA studies. In contrast, GGs with higher sialylation levels and longer glycan chains, including GD1, GT1, GQ1, GH1, GQ2, GT3, and GD2, were more commonly associated with OCFAs in prior studies on healthy brain tissue [73].

Another noteworthy observation pertains to the heterogeneity of the ceramide (Cer) moieties in the GG species expressed in BMLA. The shortest fatty acid chain (C12) was found in GM4(d18:1/12:2), GT1(d18:1/12:3), and GT1(d18:1/12:4) species, whereas the longest (C32) was detected exclusively in GM3(d18:1/32:2). Most GGs, more exactly 115 species or over 76%, contained ceramides with fatty acid chains ranging from C16 to C24. Additionally, according to the data presented in Table 1 and Figure 3, Figure 4 and Figure 5, 26 species, mainly from the GM, GT, and GD classes, contained polyunsaturated fatty acid (PUFA) moieties.

Finally, the analysis of sphingoid base composition revealed a low prevalence of trihydroxylated bases in BMLA: only 20 species (13%) contained (t18:1) or (t18:0), reflecting a significantly lower number compared with earlier IMS-MS data from C65Y tissue, where 52 species (37%) contained trihydroxylated sphingoid bases [73]. These findings support a BMLA-specific GG profile characterized by reduced complexity, altered sialylation, ceramide remodeling, and diminished OCFA and trihydroxylated base content, underscoring the potential of IMS-MS in defining metastatic glycosphingolipid signatures.

### 2.2. Structural Confirmation by Collision-Induced Dissociation Tandem MS (CID MS/MS)

**Figure 7:**
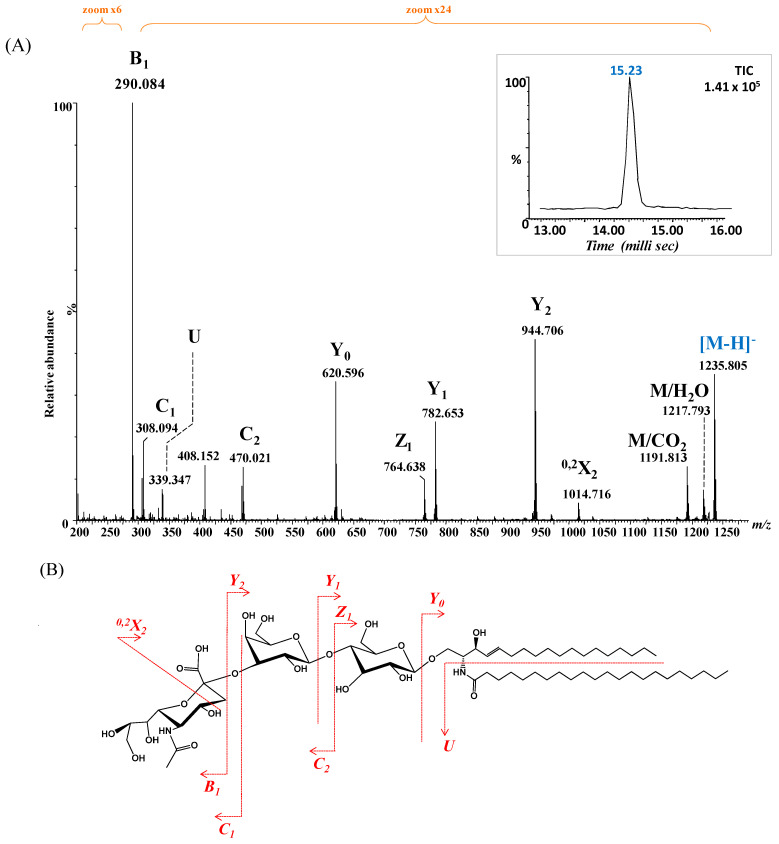
(**A**) (−) nanoESI IMS CID MS/MS of [M − H]^−^ at *m*/*z* 1235.805 corresponding to GM3(d18:1/22:0), isolated and fragmented from the BMLA sample. Inset: drift time distribution for the ion at *m*/*z* 1235.805 fragmented by CID. (**B**) Proposed fragmentation pathway of GM3(d18:1/22:0).

Given the elevated incidence of GM3 glycoforms in BLMA, and the predominance of saturated fatty acids (SFAs) over PUFAs, as reported in the Section 3, the ion detected at *m*/*z* 1235.805 (Figure 3), assigned by exact mass calculation to GM3 with a ceramide composition of (d18:1/22:0), was selected for detailed structural analysis. The precursor ion was isolated and subjected to CID fragmentation under optimized conditions, with a 2 min infusion and collision energy ramped from 40 to 65 eV, to generate diagnostic fragment ions informative for both the saccharide moiety and ceramide composition. The resulting CID MS/MS (Figure 7A), along with the fragment ion assignment and the deduced GM3 structure (Figure 7B), reveals the formation of diagnostic fragment ions.

Specifically, ions corresponding to the NeuAc monosaccharide, the NeuAc-Gal motif, detected as B_1_, C_1_ and C_2_ and *m*/*z* 290.084, *m*/*z* 308.094 and *m*/*z* 470.021, respectively, confirmed the nonreducing end. In parallel, fragment ions validating fatty acid and the entire ceramide composition (d18:1/22:0) were observed, including *m*/*z* 339.347 (fatty acid), *m*/*z* 620.596 (Cer), *m*/*z* 782.653 (Glc-Cer), and *m*/*z* 944.706 (Gal-Glc-Cer).

**Figure 8:**
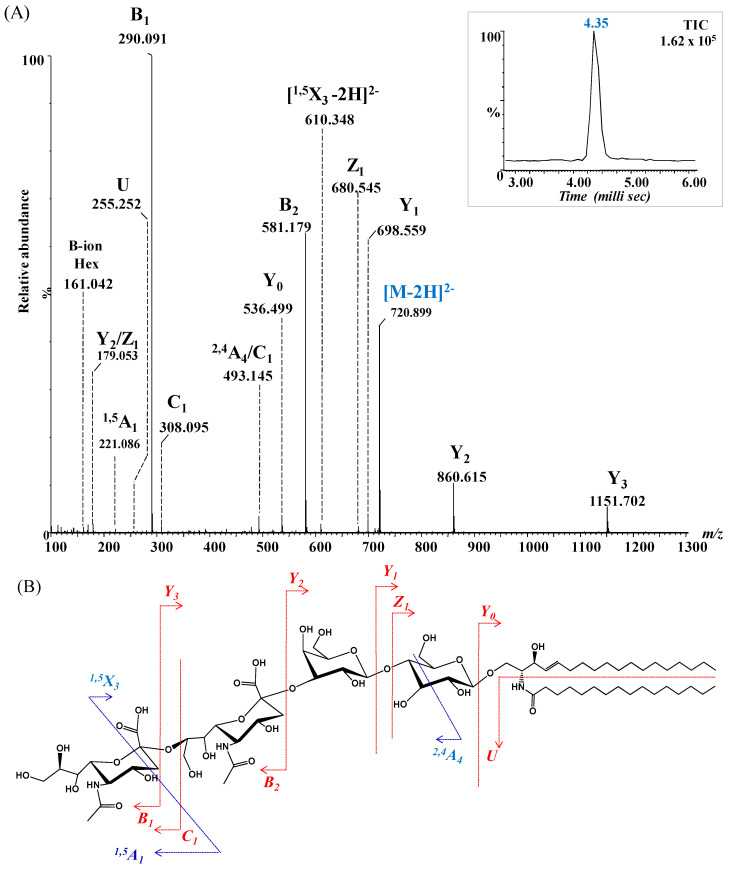
(**A**) (−) nanoESI IMS CID MS/MS of [M − 2H]^2−^ at *m*/*z* 720.899 corresponding to GD3(d18:1/16:0), isolated and fragmented from the BMLA sample. Inset: drift time distribution for the ion at *m*/*z* 720.899 fragmented by CID. (**B**) Proposed fragmentation pathway of GD3(d18:1/16:0).

To further validate the structural assignment of GG species detected by IMS-MS, CID MS/MS was also performed on the ion at *m*/*z* 720.899 (Figure 4), preliminarily assigned to GD3(d18:1/16:0) based on accurate mass measurements. Structural confirmation aimed to characterize the glycan core and ceramide composition, particularly the degree of hydroxylation and unsaturation, while minimizing desialylation during fragmentation. The resulting CID spectrum (Figure 8A), along with the proposed fragmentation pathway (Figure 8B), revealed key product ions essential for elucidating both the glycan chain and ceramide moiety.

Disialylation of the glycan chain was confirmed by the detection of characteristic fragment ions: B_1α_ at *m*/*z* 161.042 and C_1α_ at *m*/*z* 308.095, consistent with single Neu5Ac residues, and B_2α_ at *m*/*z* 581.179, representing the disialo Neu5Ac–Neu5Ac fragment. Additional diagnostic ions included Y_3α_ at *m*/*z* 1151.531 and Y_2α_ at *m*/*z* 860.615, consistent with sequential loss of sialic acids from the parent GD3 structure.

Despite relatively low intensities, ions at *m*/*z* 536.499, *m*/*z* 680.545, and *m*/*z* 698.559, assigned to Y_0_, Z_1_ and Y_1_ provided strong evidence for the presence of the Glc-Cer sequence. Furthermore, the detection of the U ion at *m*/*z* 255.252, diagnostic for palmitic acid (C16:0), conclusively confirmed the ceramide composition as (d18:1/16:0).

The fragmentation spectrum in Figure 8A reveals several internal fragments arising from cross-ring cleavages that further substantiatethe proposed structure. These included the doubly deprotonated ^1,5^X_3_ at *m*/*z* 610.348, the singly deprotonated ^1,5^A_1_ at *m*/*z* 221.086, and ^2,4^A_4_ at *m*/*z* 493.145, arising from internal fragmentation of Neu5Ac and glucose (Glc). Collectively, the fragmentation data provided robust structural confirmation for GD3(d18:1/16:0), validating both the disialylated glycan chain and its ceramide composition, and exemplifying the utility of CID MS/MS in glycosphingolipid characterization.

The fragment-ion mass errors, calculated from the absolute deviations, ranged from 1 to 13 ppm (average 6 ppm) for the fragment ions shown in Figure 7, and from 1 to 18 ppm (average 8 ppm) for those shown in Figure 8.

Notably, both fragmentation experiments yielded a single drift time (inset, Figure 7A and Figure 8A), indicating that each fragmented glycoform corresponded to a single isomer.

## 3. Discussion

BMs from LUAD represent a critical clinical challenge due to their aggressive nature, limited treatment efficacy, and poor prognosis. Despite advances in targeted therapies and immunotherapy, survival remains low because most strategies inadequately address the unique molecular adaptations that enable tumor cells to colonize the brain microenvironment.

This study provides novel insights by uncovering specific alterations in GGs within BMLA, highlighting their roles in metastatic tropism, immune evasion, and tumor progression. Through HRMS and IMS, we identified distinct molecular features that support tumor survival in the brain, advancing the understanding of lipid-based biomarkers and therapeutic targets.

Our IMS-MS investigation of the molecular mechanisms underlying BMLA tumor invasion revealed notable differences in GG expression between BMLA and C65Y [73], including a reduction in total GG species, changes in sialylation levels and alterations in ceramide chain composition.

Among the most striking changes was the marked over-expression of GM3, accounting for 21% of total GGs in BMLA. GM3 has been previously associated with various cancers [78,79], and our findings support its role in tumor progression through mechanisms such as cell adhesion, signaling, and immune evasion. Prior studies have elucidated GM3’s functional roles, highlighting its interaction with components of the basal membrane, its regulation of cellular adhesion and migration, and its participation in growth factor receptor signaling [78,79]. Moreover, GM3 has been shown to impact key intracellular signaling cascades [79]. Although its utility as a biomarker for BMs is still under investigation, our data reinforce the potential of GM3 as a tumor-associated carbohydrate antigen and suggest its promise as both a diagnostic and prognostic marker, as well as a therapeutic target in metastatic LUAD.

Additionally, elevated levels of GT1, comprising 24 distinct molecular species, were identified. GT1 is a brain-specific GG, and its enrichment in metastatic lesions points to its role in central nervous system tropism. The presence of GT1b in BMs from various carcinomas, including those of the colon, renal, lung, esophagus, pancreas, and mammary glands, as well as in primary tumors that subsequently developed BMs but not in systemic carcinomas lacking brain involvement [80], underlines the hypothesis that GT1 may serve as a predictive biomarker of brain metastasis. Moreover, the current findings lend additional support to the hypothesis that GT1 expression potentially confers an increased propensity for tumor cells to metastasize to the brain. Nevertheless, further studies are warranted to elucidate the precise role of GT1’s sialic acid moieties in facilitating the adhesion of metastatic tumor cells to brain endothelial cells and in promoting their subsequent invasion into brain parenchyma.

Conversely, asialogangliosides, undetected in healthy cerebellar tissue [73] but present in BMLA, seem to be also implicated in promoting tumor progression and metastatic spread by altering immune recognition or cell signaling [81]. Their distinctive expression patterns and structural characteristics make them attractive for diagnostic purposes, being able to monitor disease progression or response to therapy, as well as for therapeutic applications aimed at blocking metastasis or enhancing immune responses against BMLA cells.

In addition to notable alterations in sialylation patterns, our IMS-MS data revealed an unexpectedly large number of variations in the general structure of the glycan core induced by *O*-acetylation, fucosylation and modifications by CH_3_COO^−^ (Figure 5). These structural modifications are indicative of tumor metabolic reprogramming to promote survival in the brain microenvironment.

*O*-acetylation, especially of GM3 and GD3, significantly influences cancer cell survival, proliferation, invasion, and metastasis, correlating with more aggressive cancer phenotypes [82]. Considered an oncofetal marker in human tumors [82], *O*-Ac-GD3, -GD2, and -GT3 were found in melanoma [83], acute lymphoblastic leukemia [84], small cell lung carcinoma [85], glioblastoma [86], and approximately half of breast carcinomas [87]. Targeting the enzymes responsible for these modifications has shown promise in glioma treatment by regulating GG expression and function [86,88], offering potential applications in lung cancer therapy as well.

Comparative data from Figure 5 illustrate that although *O*-acetylated glycoforms were present in both BMLA and C65Y tissues, their distribution patterns differ. Specifically, 25 distinct *O*-acetylated species, such as *O*-Ac-GD3, -GM3, and -GT3, were identified in BMLA, with a notably higher frequency of acetylation across multiple GG classes compared to C65Y [73]. These findings suggest that *O*-acetylated GGs may enhance tumor cell survival by protecting gangliosides from enzymatic degradation and modulating immune recognition. Consequently, they hold promise as biomarkers for early diagnosis, prognosis, and disease monitoring in BMLA. Furthermore, their unique structural attributes and limited expression in normal tissues make them attractive targets for therapeutic interventions designed to disrupt GG-mediated signaling pathways in metastatic cancer.

Alterations in ceramide composition further underscore metabolic adaptation. Differences in ceramide chain length, saturation, and associated GG structures influence membrane fluidity and signaling [89,90]. For example, ceramides synthesized by specific ceramide synthases can either promote or inhibit tumor growth, depending on their fatty acid chain length, with certain species exhibiting pro-apoptotic properties and others conferring survival advantages [28].

Very long-chain fatty acids (VLCFAs) extend beyond the thickness of the lipid monolayer, allowing them to protrude into the cytoplasmic leaflet of the membrane bilayer, where they can exert anti-apoptotic effects [91]. In contrast, shorter ceramides more readily interact with cholesterol, influencing the organization of lipid rafts and promoting apoptotic signaling, a property lacking in longer ceramides such as C24:0, which may account for their association with enhanced cell proliferation [91]. Although fatty acid chains ranging from C12 to C26 are present in both BMLA and C65Y, their relative abundance varies, particularly with regard to the distribution of SFAs versus monounsaturated fatty acids (MUFAs) and PUFAs.

For instance, according to the literature, SFAs in BMLA contribute to reduced membrane fluidity and drug resistance, while PUFAs prevent lipid accumulation, enhance fluidity and metastasis [92,93,94]. For example, it was found that cancer cells increase SFA levels in their membranes through activated fatty acid metabolism pathways, which decreases membrane fluidity and contributes to drug resistance [94]. Hence, the higher expression in BLMA of SFAs over the PUFAs (52% versus 13%) supports the concept that cancer cells increase SFA levels through activated fatty acid metabolism pathways, which decreases membrane fluidity and contributes to drug resistance. At the same time, unsaturated fatty acids (UFAs) comprised 48% of GGs, reflecting a dual role, enhancing metastasis via increased membrane fluidity, but potentially inducing cytotoxicity at excessive levels [95,96].

In addition to the role of UFA in cancer metastasis, the specific distribution of ceramide chain lengths—particularly C16 and C18—plays a critical role in tumor behavior [28,97]. In BMLA, IMS-MS analysis revealed a notable down-regulation of pro-apoptotic C18-ceramide from 23% in C65 [73] to 15% and up-regulation of proliferative C16-ceramide, from 8% [73] to 14%. These shifts highlight a clear metabolic reprogramming favoring tumor survival and growth in metastatic brain lesions. Consistent with previous findings in head and neck squamous cell carcinoma, where decreased C18-ceramide levels correlated with malignant progression [98], this trend underscores the broader relevance of ceramide remodeling in aggressive cancers. Additionally, BMLA tissues exhibited a gradual increase in long-chain ceramides, particularly C24 species, aligning with observations by Schiffmann et al. [99] that linked elevated levels of C16-, C24:1-, and C24:0-ceramides with high-grade tumors.

Finally, rare GG species incorporating OCFAs and trihydroxylated sphingoid bases were markedly reduced in BMLA, suggesting that loss of these species may serve as an early indicator of metastatic transformation. Given the oncogenic importance of C16-ceramide and the predominance of GGs with short glycan chains in BMLA, the structure of a potential biomarker candidate was further confirmed using CID MS/MS. This approach validated the molecular identity of GD3(d18:1/16:0), a GG potentially associated with BMs proliferation.

Collectively, these molecular signatures support the hypothesis that GGs are not merely markers but active drivers of metastatic progression in LUAD. The integration of HRMS and IMS enabled precise characterization of these alterations, demonstrating the utility of advanced MS-based platforms for biomarker discovery. Although the sample size was limited, the consistency of these findings underscores their potential clinical relevance. Future studies involving larger cohorts and functional assays are required to validate the mechanistic roles of specific gangliosides and to evaluate their therapeutic potential. This work provides a foundation for the development of precision oncology strategies targeting GG-mediated pathways in LUAD brain metastasis.

## 4. Materials and Methods

### 4.1. Sampling of Brain Metastasis Originated from Lung Adenocarcinoma

This study investigated five brain metastasis tissue samples originating from LUAD, all collected from male patients aged between 70 and 80 years, with metastases located in the cerebellum. The patients exhibited comparable neurological symptoms, including sudden onset of headache, dizziness, nausea, and impaired coordination. These symptoms emerged several years after surgical resection of their primary lung tumors. In each case, neuroimaging revealed intracranial masses, most commonly located in the posterior fossa. One representative case involved a 73-year-old male patient with a hyperdense cerebellar vermis mass measuring approximately 40 × 40 × 20 mm on computed tomography. Following neurosurgical removal, pathohistological examination (Department of Neurosurgery, University Hospital, Zagreb, Croatia) revealed glandular and papillary structures lined by anaplastic columnar epithelial cells with frequent mitoses, confirming the diagnosis of brain metastasis from adenocarcinoma in all cases. After the careful excision of the blood vessels and necrotic elements, the tissue sample intended for biochemical analysis was weighed and stored at −20 °C. Informed consent was obtained from all five subjects and/or their legal guardian(s).

### 4.2. Ganglioside Extraction and Purification

GG extraction and purification were performed in the laboratories of the Faculty of Medicine, University of Zagreb, Croatia, following the method of Svennerholm and Fredman [100], and modified by Vukelić et al. [101], as described previously [23]. In brief, prior to GG extraction, each metastatic tissue was individually weighed and homogenized in ice-cold distilled water to create 10% (*w*/*v*) homogenates. Lipids were extracted twice from each sample using a solvent mixture of chloroform: methanol (1:2, by volume), followed by partition and repartition with the addition of chloroform, methanol, and water in a final volume ratio of 1:1:0.8. The upper phases, which contained the polar glycosphingolipids (gangliosides), were collected. Each crude GG extract was then purified through several steps: protein–salt complex precipitation followed by centrifugation, removal of low-molecular-weight contaminants via gel-filtration on a Sephadex G-25 column (Pharmacia, Uppsala, Sweden), and dialysis against water (overnight at 4 °C). The purified GG extracts were evaporated to dryness, weighed, and stored for further analysis. All five samples were analyzed individually.

Permission for the use of human tissues for scientific research was obtained from the Ethics Committee of the Zagreb Medical Faculty under the Project “Structure-function glycolipidomics of brain development and malignant alteration”, No.108-1081870-2415, funded by the Croatian Ministry of Science, Education and Sport. All procedures involving human tissues complied with the 1964 Helsinki Declaration and its subsequent amendments.

For nanoESI IMS-MS and CID MS/MS analyses, individual stock solutions of GG extracts (~0.5 mg/mL) were prepared for each of the five samples by dissolving the dried GG material in pure methanol and storing the solutions at −27 °C. Each stock solution was subsequently diluted in pure methanol to obtain a working aliquot with a concentration of approximately 5 pmol/μL (calculated based on an average molecular weight of 2000). Prior to MS analysis, each sample solution was centrifuged for 2 min at 6000 rpm using a mini-centrifuge (Thermo Fisher Scientific, Waltham, MA, USA). The supernatants were collected and individually subjected to (−) nanoESI IMS-MS and MS/MS analysis by CID at low energies. All reagents used in this study were purchased from Sigma (St. Louis, MO, USA) and were of analytical grade.

### 4.3. Ion Mobility Spectrometry Mass Spectrometry

Research For the IMS-MS experiments, including data acquisition and IM data processing, a Synapt G2-S mass spectrometer (Waters, Manchester, UK) equipped with a nanoESI source and interfaced with a PC running Waters MassLynx (version V4.1, SCN 855) and Waters Driftscope (version V2.7) software was used.

For each sample, a 10µL aliquot of the methanolic solution, containing GGs at a concentration of 5 pmol/µL, was introduced into the back of a 10 cm electrospray capillary as previously described [65]. A 0.25 mm platinum wire was then inserted into each solution.

Both MS and MS/MS experiments were performed in negative ion mode, within the mass range of 400–2500 *m*/*z*, at a scan rate of one scan per second, over a two-minute infusion period for each sample. The voltage applied to the platinum wire and the cone was carefully optimized to ensure efficient ionization of the components while minimizing in-source fragmentation. A stable and constant spray was achieved with a capillary voltage of 1.5 kV and a cone voltage of 45 V. Other ionization parameters were set as follows: source block temperature at 100 °C, desolvation gas flow rate at 800 or 100 L/h, and desolvation temperature at 150 °C.

The continuous ion beam generated by ESI is directed into an ion funnel, where it is converted into a pulsed beam and periodically introduced into the wave ion guide. The ions then pass through the quadrupole region and enter the drift region, which is filled with drift gas. In this region, ions are separated based on their size, charge, shape, and apparent surface area according to their mobility. To optimize GG separation, the following IMS parameters were adjusted: ion mobility gas flow at 90 mL/min, ion mobility wave velocity at 650 m/s, and ion mobility wave height at 40 V. Finally, the ions reach the TOF analyzer, operating in V-mode, where they are separated according to their *m*/*z* ratios with an average mass resolution of 20,000.

For the screening experiments, the low-mass (LM) and high-mass (HM) resolution parameters were set to 12 and 15, respectively. For CID MS/MS, these parameters were adjusted to 10 and 15, respectively. The fragmentation experiments were carried out in the transfer cell after mobility separation, using collision energies ranging from 40 to 65 eV.

To ensure the reproducibility and reliability of the IMS-MS data, each BMLA sample was analyzed in three technical replicates, thereby minimizing the impact of potential variability arising from experimental handling or instrument fluctuations. To minimize carryover between analyses, a washing step was performed after each BMLA sample run. As no meaningful differences were observed among replicates, the data displayed in the manuscript correspond to a single representative run for each sample.Under consistent nanoESI IMS-MS and MS/MS conditions, the sample-to-sample reproducibility in terms of sensitivity, number of detected molecular/fragment ions, relative intensity and charge state was 99%, while day-to-day reproducibility reached 98%. Although biological replicates (i.e., multiple samples from the same patient) were not available due to sample limitations, the consistent molecular profiles observed across the five individual patient samples further support the robustness and validity of our findings.

### 4.4. Ganglioside Abbreviation and Assignment of the Spectra

For the assignment of GGs, the abbreviation system introduced by Svennerholm (1980) [102], along with the guidelines from the IUPAC-IUB Commission on Biochemical Nomenclature (IUPAC-IUB 1998) [103], was applied.

GA1-Gg4Cer;

GA2-Gg3Cer;

GA3-LacCer;

GM1-II^3^-α-Neu5Ac-Gg_4_Cer;

GM2-II^3^-α-Neu5Ac-Gg_3_Cer;

GM3-II^3^-α-Neu5Ac-LacCer;

GM4-II^3^-α-Neu5Ac-GgCer;

GD1-II^3^-α-(Neu5Ac)_2_-Gg_4_Cer;

GD2-II^3^-α-(Neu5Ac)_2_-Gg_3_Cer;

GD3-II^3^-α-(Neu5Ac)_2_-LacCer;

GT1-II^3^-α-(Neu5Ac)_3_-Gg_4_Cer;

GT2-II^3^-α-(Neu5Ac)_3_-Gg_3_Cer;

GT3-II^3^-α-(Neu5Ac)_3_-LacCer;

GT4-II^3^-α- (Neu5Ac)_3_-GgCer;

GQ1-II^3^-α-(Neu5Ac)_4_-Gg_4_Cer;

GP2-II^3^-α-(Neu5Ac)_5_-Gg_3_Cer.

The assignment of the oligosaccharide backbone sequence ions generated during the fragmentation experiment followed the nomenclature established by Domon and Costello [104] and further revised by Costello et al. [105]. For the ceramide-related fragment ions, the nomenclature introduced by Ann and Adams was applied [106].

## 5. Conclusions

Despite advances in precision oncology, LUAD remains a major challenge due to its high risk of brain metastasis. Although molecular profiling has improved the accuracy of treatment selection, there remains a critical need for reliable biomarkers for early detection and monitoring of metastatic progression. In this context, GGs have emerged as promising candidates, with their altered expression reflecting tumor aggressiveness and serving as potential diagnostic, prognostic, and therapeutic indicators in BMLA.

In this study, the combined use of HRMS and IMS-MS enabled careful structural characterization of GGs in BMLA tissues, uncovering distinct expression signatures that distinguish metastatic from non-metastatic brain tissues. Our findings, including (i) overexpression of GM3 and brain-specific GT1 species, (ii) the presence of asialogangliosides, (iii) extensive *O*-acetylation and glycan modifications, and (iv) shifts in ceramide composition favoring pro-survival C16-ceramides and long-chain species, suggest how GG remodeling in BMLA may be associated with oncogenic processes, including tumor invasiveness, immune evasion, and survival, underscoring their potential as novel biomarkers and therapeutic targets.

Moreover, IMS-MS revealed rare lipid variants, including OCFAs and trihydroxylated sphingoid bases, emphasizing its efficacy in dissecting lipid dysregulation within the metastatic brain microenvironment. Overall, GG profiling via IMS-MS represents a powerful platform for biomarker discovery and the development of lipid-targeted therapies in oncology. Further validation of these findings in larger clinical cohorts is necessary to support their translation into precision oncology applications.

## Figures and Tables

**Table 1:** The detected and identified GG species in BMLA sample.

Ganglioside Class	Identified Species
GA1	d18:0/16:1, d18:1/18:0
GA3	d18:1/16:1, d18:1/17:0, d18:1/17:3, d18:1/17:4, d18:1/18:0, d18:1/18:0, d18:1/19:1, d18:1/20:0, d18:1/20:1, d18:1/24:0
GA2	d18:1/13:3
GM1	d18:0/14:0, d18:0/20:0, d18:1/14:0, d18:1/16:0, d18:1/16:1, d18:1/16:2, d18:1/18:0, d18:1/18:1, d18:1/18:2, d18:1/20:0, d18:1/20:2, d18:1/21:1, d18:1/22:0, d18:1/22:1, d18:1/22:2, d18:1/24:1, t18:1/16:0, t18:1/18:1, *O*-Ac-d18:1/16:0, *O*-Ac-d18:1/20:0, *O*-Ac-d18:1/20:1, (CH_3_COO^−^)-d18:0/18:0
GM2	d18:1/16:3, d18:1/18:1, d18:1/20:0
GM3	d18:0/18:0, d18:0/24:0, d18:1/14:0, d18:1/16:0, d18:1/16:1, d18:1/16:4, d18:1/18:0, d18:1/18:1, d18:1/19:0, d18:1/19:4, d18:1/20:0, d18:1/20:1, d18:1/22:0, d18:1/22:1, d18:1/23:0, d18:1/23:3, d18:1/24:0, d18:1/24:1, d18:1/32:2, t18:1/14:0, t18:1/14:2, t18:1/16:0, t18:1/16:1, t18:1/18:0, t18:1/20:0, t18:1/20:1, t18:1/22:0, t18:1/22:1, t18:1/24:0, *O*-Ac-d18:0/20:0, *O*-Ac-d18:1/20:0, di-*O*-Ac-d18:0/18:0, di-*O*-Ac-d18:0/22:0
GM4	d18:1/12:2, d18:1/14:0, d18:1/16:2
GD1	d18:0/20:0, d18:0/24:0, d18:1/16:0, d18:1/18:0, d18:1/18:1, d18:1/20:0, d18:1/21:0, d18:1/22:0, d18:1/22:2, d18:1/23:1, d18:1/24:0, d18:1/24:1, t18:0/16:0, t18:0/18:0, t18:0/20:0, t18:1/16:0, t18:1/20:1, t18:1/22:2, *O*-Ac-d18:0/20:0, *O*-Ac-d18:1/20:0, *O*-Ac-d18:1/20:1, *O*-Ac-d18:1/22:0, Fuc-t18:0/16:0, (CH_3_COO^−^)-d18:1/19:0
GD2	d18:0/20:0, d18:1/16:2, d18:1/18:0, d18:1/20:0, d18:1/20:1, d18:1/20:2, d18:1/22:2
GD3	d18:1/16:0, d18:1/18:0, d18:1/24:2, d18:1/26:0, d18:1/26:2, *O*-Ac-d18:0/26:0, *O*-Ac-d18:1/18:0, *O*-Ac-d18:1/26:0
GT1	d18:0/21:0, d18:1/12:3, d18:1/12:4, d18:1/16:0, d18:1/18:0, d18:1/18:1, d18:1/20:0, d18:1/20:3, d18:1/22:0, d18:1/22:1, d18:1/22:3, d18:1/26:1, d18:1/29:2, t18:1/18:0, t18:1/20:0, t18:1/20:1, t18:1/20:1, t18:1/22:1, *O*-Ac-d18:0/20:0, *O*-Ac-d18:0/22:0, *O*-Ac-d18:1/18:2, d18:1/20:2, *O*-Ac-d18:1/22:2, Fuc-d18:0/16:0, Fuc-d18:1/16:0
GT2	d18:1/23:1
GT3	d18:0/16:0, d18:1/24:1, t18:0/20:0, *O*-Ac-d18:1/18:1, *O*-Ac-d18:1/22:0, *O*-Ac-d18:1/24:0, Fuc-d18:1/20:1, Fuc-t18:1/16:0
GQ1	d18:1/12:0, d18:1/14:2, d18:1/18:0, d18:1/20:0, d18:1/24:0, d18:1/24:2, *O*-Ac-d18:1/18:0, *O*-Ac-d18:1/22:0
GP2	d18:1/24:2

d-dihydroxylatedsphingoid base; t-trihydroxylatedsphingoid base.

## Data Availability

The original contributions presented in this study are included in the article/Supplementary Material. Further inquiries can be directed to the corresponding author(s).

## Supplementary Materials

The following supporting information can be downloaded at: https://www.mdpi.com/article/10.3390/ijms262412029/s1.

## Abbreviations

The following abbreviations are used in this manuscript:

BBB	blood–brain barrier
BM	brain metastases
BMLA	brain metastases of lung adenocarcinoma
Cer	ceramide
CID MS/MS	collision-induced dissociation tandem MS
CT	computed tomography
GG	ganglioside
HM	high resolution
HRMS	high-resolution mass spectrometry
ICI	immune checkpoint inhibitor
IMS	ion mobility spectrometry
IMS-MS	ion mobility spectrometry mass spectrometry
LM	low resolution
LUAD	lung adenocarcinoma
MRI	magnetic resonance imaging
MS	mass spectrometry
MUFA	monounsaturated fatty acid
nanoESI	nanoelectrospray ionization
Neu5Ac	sialic acid/N-Acetylneuraminic acid
NSCLC	non-small cell lung carcinoma
OCFA	odd-chained fatty acid
PUFA	polyunsaturated fatty acid
SCLC	small cell lung carcinoma
SFA	saturated fatty acid
TKI	tyrosine kinase inhibitor
UFA	unsaturated fatty acid
VLCFA	very long-chain fatty acids
