# Supplementary material for: Overexpression of GM3 and Ganglioside Pattern Remodeling in Lung Adenocarcinoma Brain Metastases Identified by Ion Mobility Mass Spectrometry
Source: Int J Mol Sci. 2025 Dec 14;26(24):12029. doi: 10.3390/ijms262412029 (PMC12732680; doi:10.3390/ijms262412029)
Supplement: Supplementary file 1 [file ijms-26-12029-s001.zip › ijms-3985089-supplementary.pdf]

International Journal of
Molecular Sciences

MDPI

Article

Overexpression of GM3 and Ganglioside Pattern Remodeling in Lung Adenocarcinoma Brain Metastases Identified by Ion Mobility Mass Spectrometry

Mirela Sarbu [1], Raluca Ica [1,2], Željka Vukelić [3], David E. Clemmer [4] and Alina D. Zamfir [2,*]

[1] Department of Condensed Matter, National Institute for Research and Development in Electrochemistry and Condensed Matter, Timisoara, Romania; mirela.sarbu86@yahoo.co.uk
[2] Department of Technical and Natural Sciences, "Aurel Vlaicu" University of Arad; raluca.ica@gmail.com, alina.zamfir@uav.ro
[3] Department of Chemistry and Biochemistry, Faculty of Medicine, University of Zagreb, Croatia; zeljka.vukelic@mef.hr
[3] Department of Chemistry, The College of Arts & Science, Indiana University, Bloomington, Indiana, USA;clemmer@iu.edu
* Correspondence: alina.zamfir@uav.ro; Tel.: +40-256-494413

Table S1. Assignment of major ionic species detected in the BMLA ganglioside mixture by (–) nanoESI IMS-MS, including *m/z* values and mass errors. Putative species (#) indicate assignments lacking independent structural confirmation.

m/z exp	*m/z* theor	Proposed structure*	Molecular Ion	Mass accuracy (ppm)
428.791	428.797	GA3(d18:1/16:1) #	[M-2H]$^{2-}$	14.0
432.776	432.781	GA3(d18:1/17:4) #	[M-2H]$^{2-}$	11.6
433.793	433.789	GA3(d18:1/17:3) #	[M-2H]$^{2-}$	9.2
436.817	436.813	GA3(d18:1/17:0) #	[M-2H]$^{2-}$	9.2
443.828	443.821	GA3(d18:1/18:0)	[M-2H]$^{2-}$	15.8
449.817	449.821	GA3(d18:1/19:1)	[M-2H]$^{2-}$	8.9
456.822	456.829	GA3(d18:1/20:1)	[M-2H]$^{2-}$	15.4
457.830	457.837	GA3(d18:1/20:0)	[M-2H]$^{2-}$	15.3
464.285	464.279	GM4(d18:1/12:2)	[M-2H]$^{2-}$	12.9
468.835	468.828	GA3(d18:1/20:0)	[M-3H+Na]$^{2-}$	15.0
480.317	480.311	GM4(d18:1/14:0) #	[M-2H]$^{2-}$	12.5
492.318	492.310	GM4(d18:1/16:2) #	[M-2H]$^{2-}$	16.3
496.865	496.859	GA3(d18:1/24:0)	[M-3H +Na]$^{2-}$	12.1
507.301	507.293	GA2(d18:1/13:3) #	[M-2H]$^{2-}$	15.8
510.252	510.254	GM1(d18:1/14:0) #	[M-5H+2Na]$^{3-}$	3.9
510.921	510.926	GM1(d18:0/14:0) #	[M-5H+2Na]$^{3-}$	9.8
518.923	518.926	GM1(d18:1/16:1)	[M-5H+2Na]$^{3-}$	5.8
520.274	520.270	GM1(d18:1/18:2)	[M-4H+Na]$^{3-}$	7.7

520.944	520.942	GM1(d18:1/18:1)	$[M\text{-}4H\text{+}Na]^{3-}$	3.8
523.630	523.633	GM1(d18:1/20:0)	$[M\text{-}3H]^{3-}$	5.7
524.299	524.303	GM1(d18:0/20:0)	$[M\text{-}3H]^{3-}$	7.6
526.303	526.299	GM1(d18:1/22:1)	$[M\ \text{-}3H]^{3-}\text{-}H_2O$	7.6
528.945	528.942	GM1(d18:1/18:0)	$[M\text{-}5H\text{+}2Na]^{3-}$	5.7
530.961	530.958	GM1(d18:1/20:0)	$[M\text{-}4H\text{+}Na]^{3-}$	5.7
531.631	531.630	GM1(d18:0/20:0)	$[M\text{-}4H\text{+}Na]^{3-}$	1.9
536.963	536.962	*O*-Ac-GM1(d18:1/20:1)	$[M\text{-}3H]^{3-}$	1.9
537.636	537.634	*O*-Ac-GM1(d18:1/20:0)	$[M\text{-}3H]^{3-}$	3.7
540.982	540.987	*O*-Ac-GD3(d18:1/26:0) #	$[M\text{-}3H]^{3-}$	9.3
541.663	541.659	*O*-Ac-GD3(d18:0/26:0) #	$[M\text{-}3H]^{3-}$	7.4
545.611	545.613	GM1(d18:1/20:0)	$[M\text{-}6H\text{+}3Na]^{3-}$	3.7
546.615	546.613	GD2(d18:1/16:2) #	$[M\text{-}3H^+]^{3-}$	3.7
557.306	557.301	GD2(d18:1/18:0)	$[M\text{-}3H]^{3-}$	9.0
559.298	559.297	GD2(d18:1/20:2)	$[M\text{-}H_2O\text{-}3H^+]^{3-}$	1.8
559.965	559.969	GD2(d18:1/20:1)	$[M\text{-}H_2O\text{-}3H]^{3-}$	7.2
560.639	560.641	GD2(d18:1/20:0)	$[M\text{-}H_2O\text{-}3H]^{3-}$	3.6
561.319	561.313	GD2(d18:0/20:0)	$[M\text{-}H_2O\text{-}3H]^{3-}$	10.7
565.305	565.301	GD2(d18:1/20:2)	$[M\text{-}3H]^{3-}$	7.1
599.347	599.351	GM3(d18:1/18:1)	$[M\text{-}3H\text{+}Na]^{2-}$	6.7
602.381	602.376	GM3(d18:1/20:1)	$[M\text{-}2H]^{2-}$	8.3
613.374	613.368	GM3(d18:1/20:1)	$[M\text{-}3H\text{+}Na]^{2-}$	10.6
613.382	613.380	GA1(d18:0/16:0)	$[M\text{-}2H]^{2-}$	4.1
616.395	616.392	GM3(d18:1/22:1)	$[M\text{-}2H]^{2-}$	4.9
617.406	617.400	GM3(d18:1/22:0)	$[M\text{-}2H]^{2-}$	9.7
621.369	621.365	GM3(t18:1/20:1)	$[M\text{-}3H\text{+}Na]^{2-}$	6.4
621.394	621.387	GM3(d18:1/23:3) #	$[M\text{-}2H]^{2-}$	11.3
624.391	624.389	*O*-Ac-GM3(d18:1/20:0)	$[M\text{-}2H]^{2-}$	3.2
624.411	624.408	GM3(d18:1/23:0) #	$[M\text{-}2H]^{2-}$	4.8
630.410	630.407	GM3(d18:1/24:1)	$[M\text{-}2H]^{2-}$	4.8
632.389	632.386	di-*O*-Ac-GM3(d18:0/18:0) #	$[M\text{-}2H]^{2-}$	4.7
660.422	660.418	di-*O*-Ac-GM3(d18:0/22:0) #	$[M\text{-}2H]^{2-}$	6.1
661.398	661.395	GM3(t18:1/24:0) #	$[M\text{-}4H\text{+}2Na]^{2-}$	4.5
673.870	673.868	GM2(d18:1/16:3)	$[M\text{-}2H]^{2-}$	3.0
677.021	677.017	GT2(d18:1/23:1) #	$[M\text{-}3H]^{3-}$	5.9
689.902	689.900	GM2(d18:1/18:1)	$[M\text{-}2H]^{2-}$	2.9
699.006	699.007	GT1(d18:1/16:0)	$[M\text{-}3H]^{3-}$	1.4
707.677	707.678	GT1(d18:1/18:1)	$[M\text{-}3H]^{3-}$	1.4
708.348	708.350	GT1(d18:1/18:0)	$[M\text{-}3H]^{3-}$	2.8
717.693	717.694	GT1(d18:1/20:0)	$[M\text{-}3H]^{3-}$	1.4
720.899	720.900	GD3(d18:1/16:0)	$[M\text{-}2H]^{2-}$	1.4

722.351	722.354	GT1(t18:1/20:1)	$[M-3H]^{3-}$	4.2
723.029	723.026	GT1(t18:1/20:0)	$[M-3H]^{3-}$	4.1
723.039	723.037	GT1(d18:0/21:0)	$[M-3H]^{3-}$	2.8
726.370	726.366	GT1(d18:1/22:1)	$[M-3H]^{3-}$	5.5
727.037	727.038	GT1(d18:1/22:0)	$[M-3H]^{3-}$	1.4
731.695	731.698	GT1(t18:1/22:1)[#]	$[M-3H]^{3-}$	4.1
734.915	734.916	GD3(d18:1/18:0)	$[M-2H]^{2-}$	1.4
735.708	735.710	*O*-Ac-GT1(d18:0/22:0)	$[M-H_2O-3H]^{3-}$	2.7
757.918	757.919	GM1(d18:1/16:0)	$[M-2H]^{2-}$	1.3
758.391	758.394	GT1(d18:1/29:2)[#]	$[M-3H^+]^{3-}$	4.0
766.891	766.894	GM1(d18:1/16:2)	$[M-3H+Na]^{2-}$	3.9
771.935	771.934	GM1(d18:1/18:0)	$[M-2H]^{2-}$	1.3
774.945	774.947	GD3(d18:1/24:2)	$[M-2H]^{2-}$	2.6
777.345	777.348	GQ1(d18:1/12:0)	$[M-3H]^{3-}$	3.9
778.919	778.924	*O*-Ac-GM1(d18:1/16:0)	$[M-2H]^{2-}$	6.4
778.922	778.920	GM1(t18:1/18:1)	$[M-2H]^{2-}$	2.6
785.352	785.348	GQ1(d18:1/14:2)[#]	$[M-3H]^{3-}$	5.1
788.970	788.963	GD3(d18:1/26:2)[#]	$[M-2H]^{2-}$	8.9
797.945	797.950	GM1(d18:1/22:2)	$[M-2H]^{2-}$	6.3
797.948	797.949	GM1(d18:0/20:0)	$[M-3H +Na]^{2-}$	1.3
805.384	805.382	GQ1(d18:1/18:0)	$[M-3H]^{3-}$	2.5
805.911	805.916	GM1(d18:1/20:2)	$[M-4H+2Na]^{2-}$	6.2
806.952	806.955	*O*-Ac-GM1(d18:1/20:0)	$[M-2H]^{2-}$	3.7
814.724	814.726	GQ1(d18:1/20:0)	$[M-3H]^{3-}$	2.5
832.737	832.741	GQ1(d18:1/24:2)	$[M-3H]^{3-}$	4.8
836.453	836.455	GD2(d18:1/18:0)	$[M-2H]^{2-}$	2.4
862.475	862.471	GD2(d18:1/22:2)	$[M-2H]^{2-}$	4.6
875.086	875.082	GP2(d18:1/24:2)[#]	$[M-3H]^{3-}$	4.6
878.449	878.447	GT3(d18:0/16:0)	$[M-3H+Na]^{2-}$	2.3
900.457	900.460	*O*-Ac-GT3(d18:1/18:1)	$[M-2H]^{2-}$	3.3
903.468	903.466	GD1(d18:1/16:0)	$[M-2H]^{2-}$	2.2
903.481	903.485	GT3(t18:0/20:0)	$[M-2H]^{2-}$	4.4
907.463	907.468	GD1(d18:1/18:1)	$[M-H_2O-2H]^{2-}$	5.5
911.465	911.464	GD1(t18:1/16:0)	$[M-2H]^{2-}$	1.1
917.483	917.482	GD1(d18:1/18:0)	$[M-2H]^{2-}$	1.1
921.504	921.502	GT3(d18:1/24:1)[#]	$[M-2H]^{2-}$	2.2
927.468	927.465	GD1(d18:1/18:1)	$[M-3H+Na]^{2-}$	3.2
931.491	931.498	GD1(d18:1/20:0)	$[M-2H]^{2-}$	7.5
	931.485	*O*-Ac-GT3(d18:1/22:0)	$[M-H_2O-3H+Na]^{2-}$	6.4
938.492	938.501	GD1(d18:1/21:0)	$[M-2H^+]^{2-}$	9.6
	938.483	GD1(t18:1/20:1)	$[M-2H^+]^{2-}$	9.6

940.507	940.503	GD1(t18:0/20:0)	$[M-2H]^{2-}$	4.3
943.502	943.497	GD1(d18:0/20:0)	$[M-3H+Na]^{2-}$	5.3
	943.497	GD1(d18:1/22:2)	$[M-2H]^{2-}$	5.3
945.508	945.513	GD1(d18:1/22:0)	$[M-2H]^{2-}$	5.3
	945.501	*O*-Ac-GT3(d18:1/24:0)	$[M-H_2O-3H+Na]^{2-}$	7.4
948.464	948.468	GD1(t18:0/18:0)	$[M-4H^{+}+2Na^{+}]^{2-}$	4.2
951.498	951.495	*O*-Ac-GD1(d18:1/20:1)	$[M-2H]^{2-}$	3.2
	951.491	GD1(t18:1/22:2)	$[M-2H^{+}]^{2-}$	7.4
	951.509	GD1(d18:1/23:1)	$[M-2H^{+}]^{2-}$	11.6
952.499	952.503	*O*-Ac-GD1(d18:1/20:0)	$[M-2H]^{2-}$	4.2
953.513	953.511	*O*-Ac- GD1(d18:0/20:0)	$[M-2H]^{2-}$	2.1
958.461	958.465	Fuc-GT3(t18:1/16:0) #	$[M-3H+Na]^{2-}$	4.2
958.517	958.521	GD1(d18:1/24:1)	$[M-2H]^{2-}$	4.2
959.526	959.529	GD1(d18:1/24:0)	$[M-2H]^{2-}$	3.1
960.540	960.537	GD1(d18:0/24:0)	$[M-2H]^{2-}$	3.1
965.485	965.491	(CH_3COO^{-}) GD1(d18:1/19:0) #	$[M^{-}-2H+Na]^{2-}$	6.2
966.510	966.500	Fuc-GT3(d18:1/20:1)	$[M-2H]^{2-}$	10.4
	966.518	*O*-Ac-GD1(d18:1/22:0)	$[M-2H]^{2-}$	8.3
985.506	985.500	Fuc-GD1(t18:0/16:0) #	$[M-2H]^{2-}$	6.1
1008.947	1008.953	GT1(d18:1/12:3) #	$[M-2H]^{2-}$ $-H_2O$	6.0
1016.944	1016.950	GT1(d18:1/12:4) #	$[M-2H]^{2-}$	5.9
1063.027	1063.029	GT1(d18:1/18:0)	$[M-2H]^{2-}$	1.9
1074.017	1074.021	GT1(d18:1/20:3) #	$[M-2H]^{2-}$	3.7
	1074.020	GT1(d18:1/18:0)	$[M-3H+Na]^{2-}$	2.8
1077.042	1077.045	GT1(d18:1/20:0)	$[M-2H]^{2-}$	2.8
1082.015	1082.018	*O*-Ac-GT1(d18:1/18:2) #	$[M-2H]^{2-}$	2.8
	1082.018	GT1(t18:1/18:0)	$[M-3H+Na]^{2-}$	2.8
1088.033	1088.037	GT1(d18:1/22:3) #	$[M-2H]^{2-}$	3.7
1091.062	1091.061	GT1(d18:1/22:0)	$[M-2H]^{2-}$	0.9
1095.024	1095.026	GT1(t18:1/20:1)	$[M-3H+Na]^{2-}$	1.8
1095.029	1095.026	*O*-Ac-GT1(d18:0/20:0)	$[M-2H]^{2-}$	2.7
1107.029	1107.025	*O*-Ac-GT1(d18:1/20:2)	$[M-3H+Na]^{2-}$	2.7
1110.047	1110.050	*O*-Ac-GT1(d18:1/22:2)	$[M-2H]^{2-}$	2.7
1118.079	1118.080	GT1(d18:1/26:1) #	$[M-2H]^{2-}$	0.9
1122.047	1122.039	Fuc-GT1(d18:1/16:0)	$[M-2H]^{2-}$	7.1
1123.055	1123.046	Fuc-GT1(d18:0/16:0)	$[M-2H]^{2-}$	8.0
1123.685	1123.682	GM3(d18:1/14:0)	$[M-H]^{-}$	2.7
1135.648	1135.645	GM3(t18:1/14:2) #	$[M-H]^{-}$	2.6
1139.675	1139.677	GM3(t18:1/14:0) #	$[M-H]^{-}$	1.8
1143.646	1143.642	GM3(d18:1/16:4) #	$[M-H]^{-}$	-3.5

1149.696	1149.697	GM3(d18:1/16:1)	$[M-H]^-$	0.9
1151.711	1151.713	GM3(d18:1/16:0)	$[M-H]^-$	1.7
1165.687	1165.692	GM3(t18:1/16:1) [#]	$[M-H]^-$	4.3
1167.702	1167.708	GM3(t18:1/16:0) [#]	$[M-H]^-$	5.1
1179.743	1179.744	GM3(d18:1/18:0)	$[M-H]^-$	0.8
1181.758	1181.760	GM3(d18:0/18:0)	$[M-H]^-$	1.7
1185.679	1185.688	GM3(d18:1/19:4) [#]	$[M-H]^-$	-8.1
1193.761	1193.7511	GM3(d18:1/19:0) [#]	$[M-H]^-$	8.2
1195.735	1195.739	GM3(t18:1/18:0)	$[M-H]^-$	3.3
1199.569	1199.571	GQ1(d18:1/18:0)	$[M-2H]^{2-}-H_2O$	1.7
1207.774	1207.776	GM3(d18:1/20:0)	$[M-H]^-$	1.7
1221.754	1221.755	GM3(t18:1/20:1)	$[M-H]^-$	0.8
1223.768	1223.771	GM3(t18:1/20:0)	$[M-H]^-$	2.5
1235.805	1235.807	GM3(d18:1/22:0)	$[M-H]^-$	1.6
1240. 570	1240.573	*O*-Ac-GQ1(d18:1/18:0)	$[M-3H+Na]^{2-}$	2.4
1249.783	1249.786	GM3(t18:1/22:1) [#]	$[M-H]^-$	2.4
	1249.786	*O*-Ac-GM3(d18:1/20:0)	$[M-H]^-$	2.4
1251.798	1251.802	GM3(t18:1/22:0)	$[M-H]^-$	3.2
	1251.802	*O*-Ac-GM3(d18:0/20:0)	$[M-H]^-$	3.2
1253.778	1253.781	GA1(d18:1/18:0)	$[M-H]^-$	2.4
1261.819	1261.822	GM3(d18:1/24:1)	$[M-H]^-$	2.4
1263.837	1263.838	GM3(d18:1/24:0)	$[M-H]^-$	0.8
1264.845	1264.846	GM3(d18:0/24:0)	$[M-H]^-$	0.8
1268.601	1268.604	*O*-Ac-GQ1(d18:1/22:0)	$[M-3H+Na]^{2-}$	2.4
1272.603	1272.606	GQ1(d18:1/24:0)	$[M-4H+2Na]^{2-}$	2.4
1275.761	1275.763	GA1(d18:1/18:0)	$[M-2H+Na]^-$	1.6
1297.742	1297.745	GA1(d18:1/18:0)	$[M-3H+2Na]^-$	2.3
1371.929	1371.922	GM3(d18:1/32:2) [#]	$[M-H]^-$	4.6
1410.860	1410.855	GM2(d18:1/20:0)	$[M-H]^-$	3.5
1442.801	1442.808	GD3(d18:1/16:0)	$[M-H]^-$	4.9
1512.842	1512.849	*O*-Ac-GD3(d18:1/18:0)	$[M-H]^-$	4.6
1516.843	1516.845	GM1(d18:1/16:0)	$[M-H]^-$	1.3
1532.838	1532.840	GM1(t18:1/16:0)	$[M-H]^-$	1.3
1600.938	1600.939	GM1(d18:1/22:0)	$[M-H]^-$	0.6
1626.942	1626.954	GM1(d18:1/24:1)	$[M-H]^-$	7.4
	1626.929	GD3(d18:1/26:0)	$[M-2H+2Na]^-$	8.0
1628.881	1628.872	GM1(d18:1/21:1)	$[M-3H+2Na]^-$	5.5
	1628.895	(CH_3COO^-) GM1(d18:0/18:0)	$[M^- -H+Na]^-$	8.6
1807.945	1807.940	GD1(d18:1/16:0)	$[M-H]^-$	2.8
1825.948	1825.951	GD1(t18:0/16:0)	$[M-H]^-$	1.6

d-dihydroxylated sphingoid base; t-trihydroxylated sphingoid base.

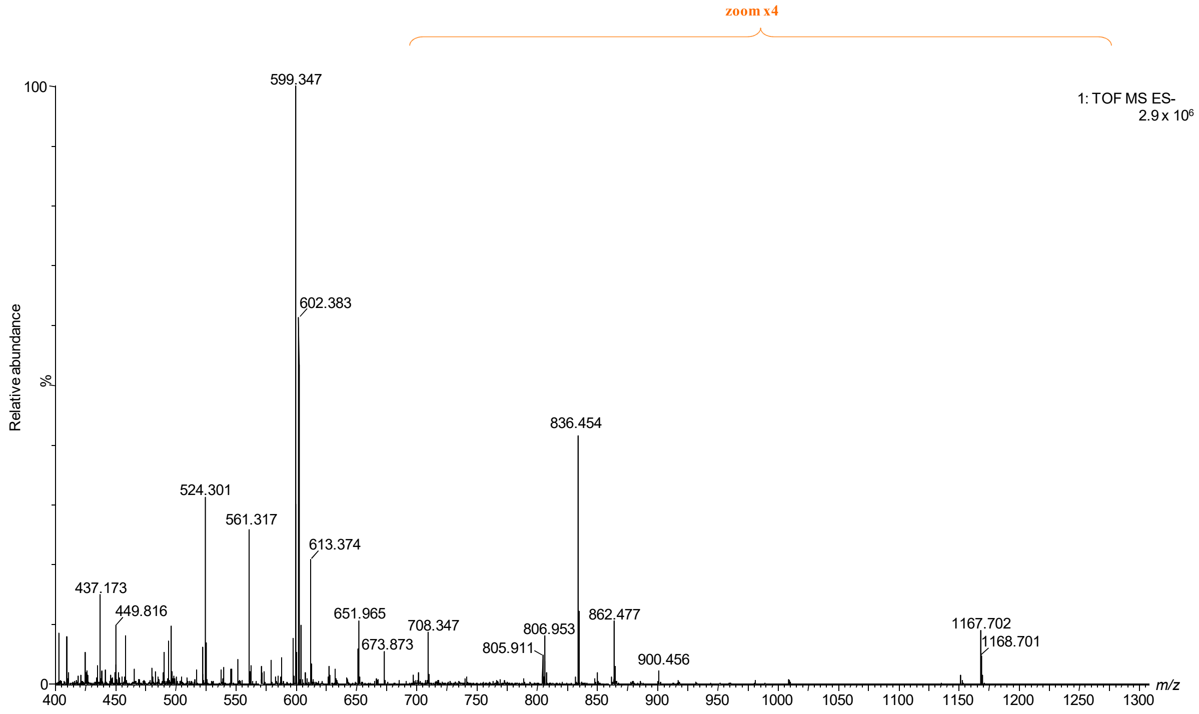

Figure S1. Full (–) nanoESI IMS-MS of BMLA2.

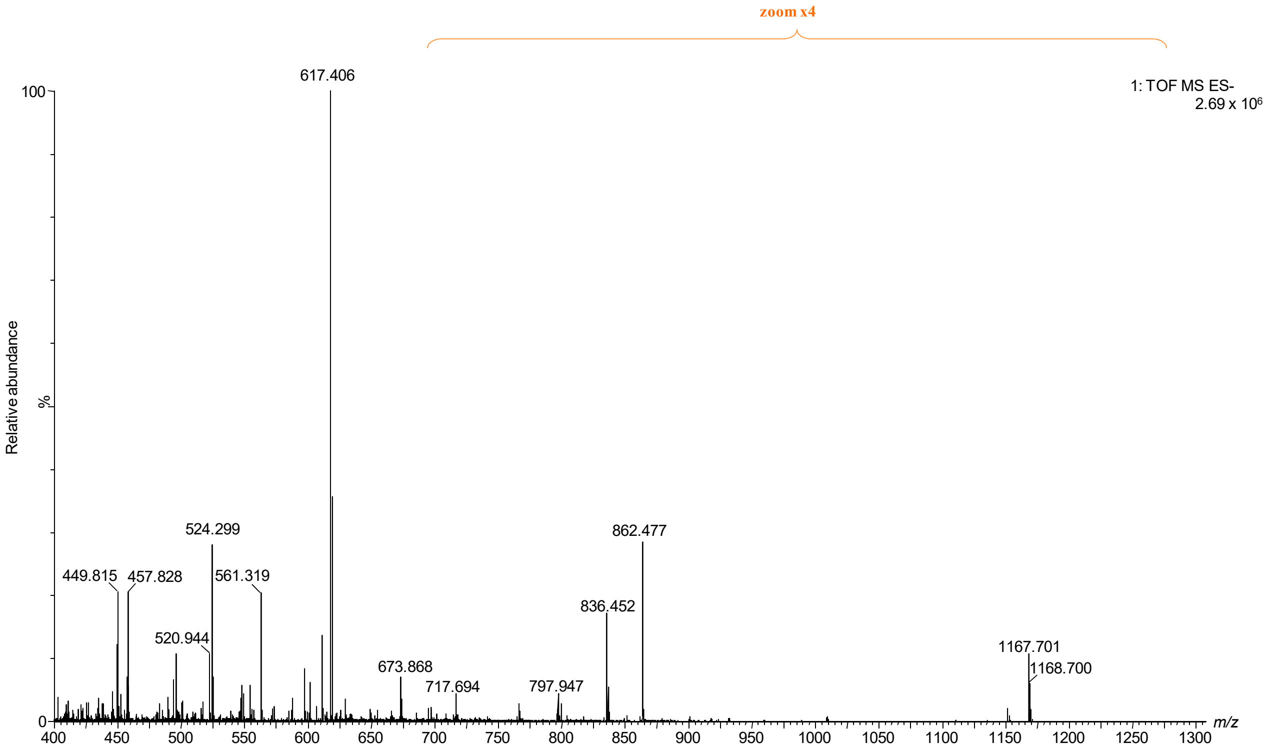

Figure S2. Full (–) nanoESI IMS-MS of BMLA3.

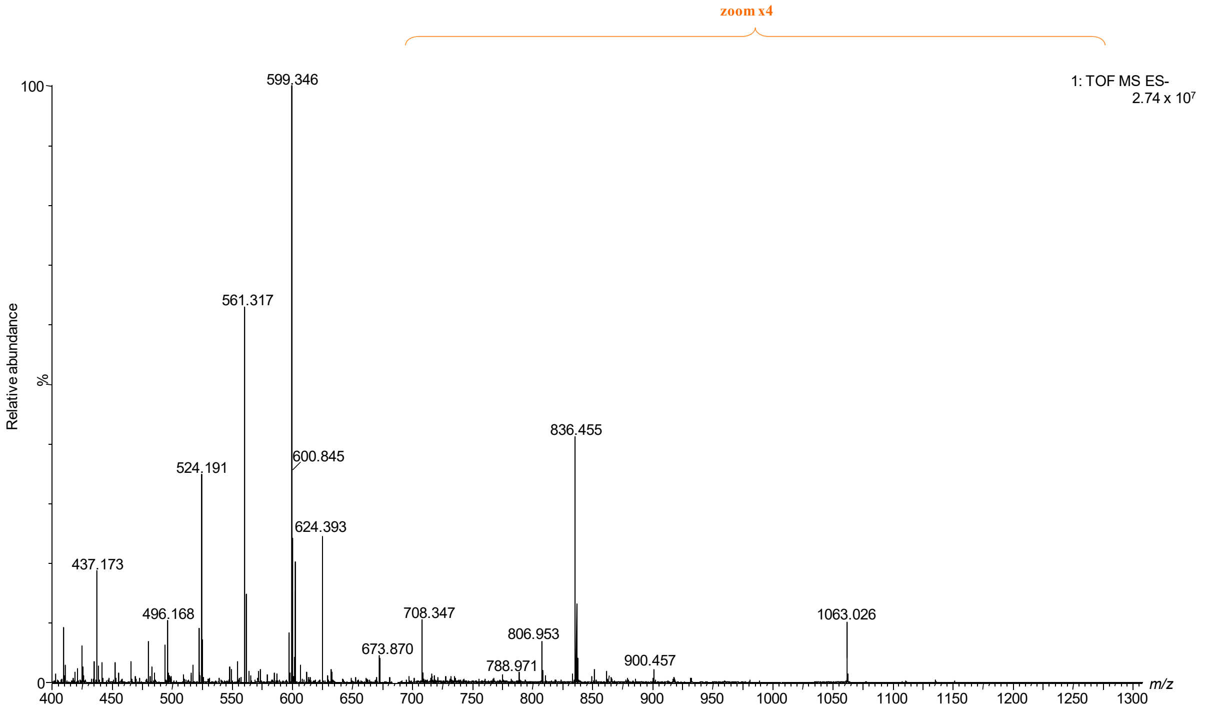

Figure S3. Full (–) nanoESI IMS-MS of BMLA4.

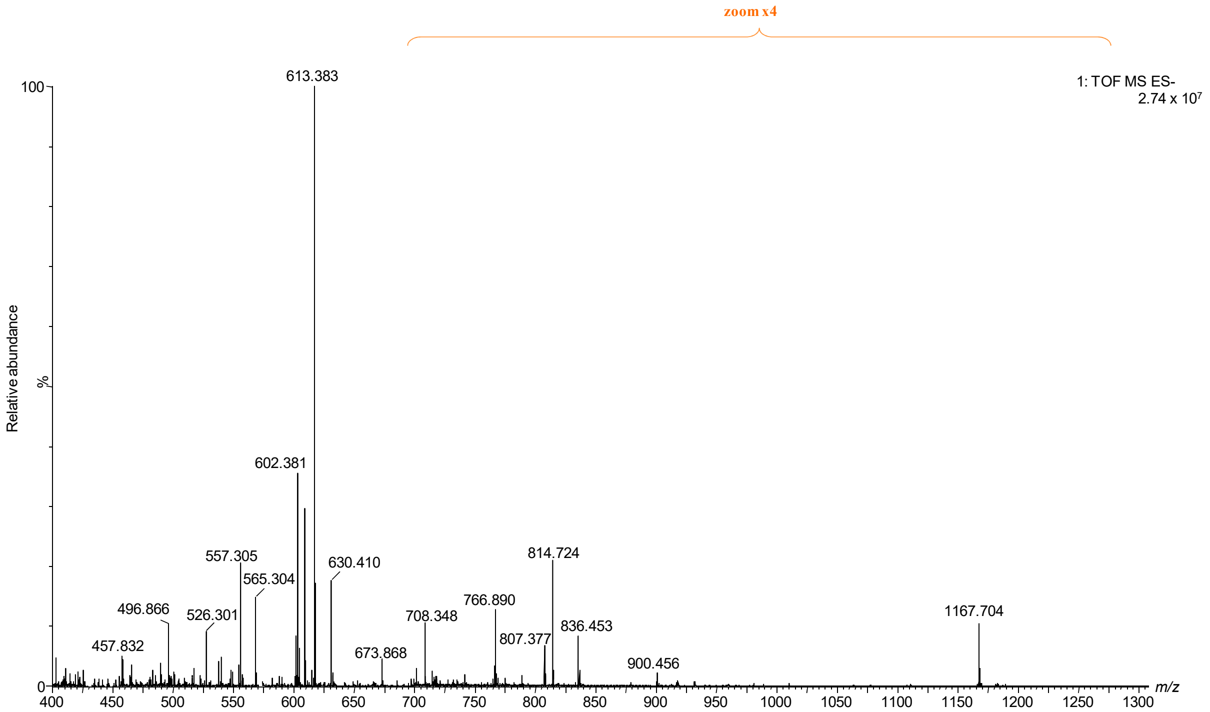

Figure S4. Full (–) nanoESI IMS-MS of BMLA5.
